# Supplementary material for: Alteration in tyrosine phosphorylation of cardiac proteome and EGFR pathway contribute to hypertrophic cardiomyopathy
Source: Commun Biol. 2022 Nov 15;5:1251. doi: 10.1038/s42003-022-04021-4 (PMC9666710; doi:10.1038/s42003-022-04021-4)
Supplement: Supplementary file 2 — Supplementary Information [file 42003_2022_4021_MOESM2_ESM.pdf]

Description of Supplementary Material Files

Supplementary Figures:

Supplementary Figure 1.

Supplementary Figure 2.

Supplementary Figure 3.

Supplementary Figure 4.

Supplementary Figure 5.

Supplementary Figure 6.

Supplementary Data 1

Supplementary Data 2

Supplementary Data 3

Supplementary Data 4

Supplementary Data 5

Supplementary Data 6

Supplementary Data 7

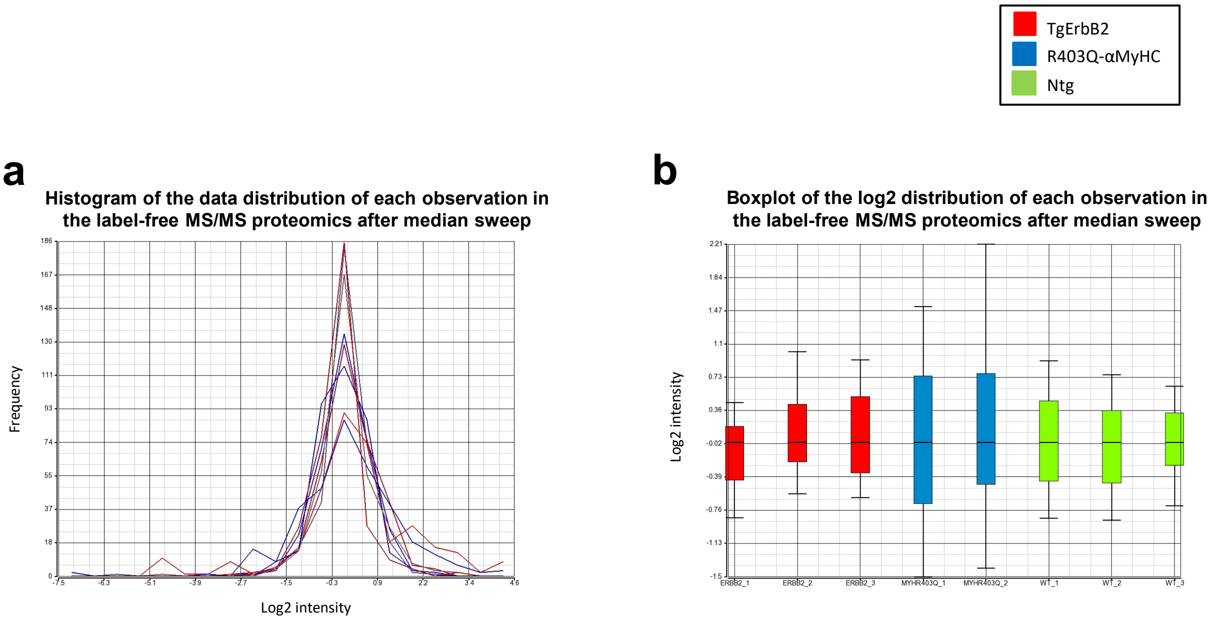

Supplementary Figure 1. Distribution of the label-free data set after median sweep normalization. a Histogram showing the data distribution of the phosphoproteome log2 intensities of each observation in the label-free MS/MS proteomics. Each line represent one observation and the values represented are the log2 intensity after median subtraction (y-axis) vs frequency. **b** Boxplot showing the data distribution per observation, the y-axis represents the phosphoproteome log2 intensity from each observation. The colors indicate the group to which each observation belong, TgErbB2 (red), R403Q-αMyHC (blue), and Ntg (green). Notice in both plots that the median of the data is equal to 0. This method retains the spreading of the original data.

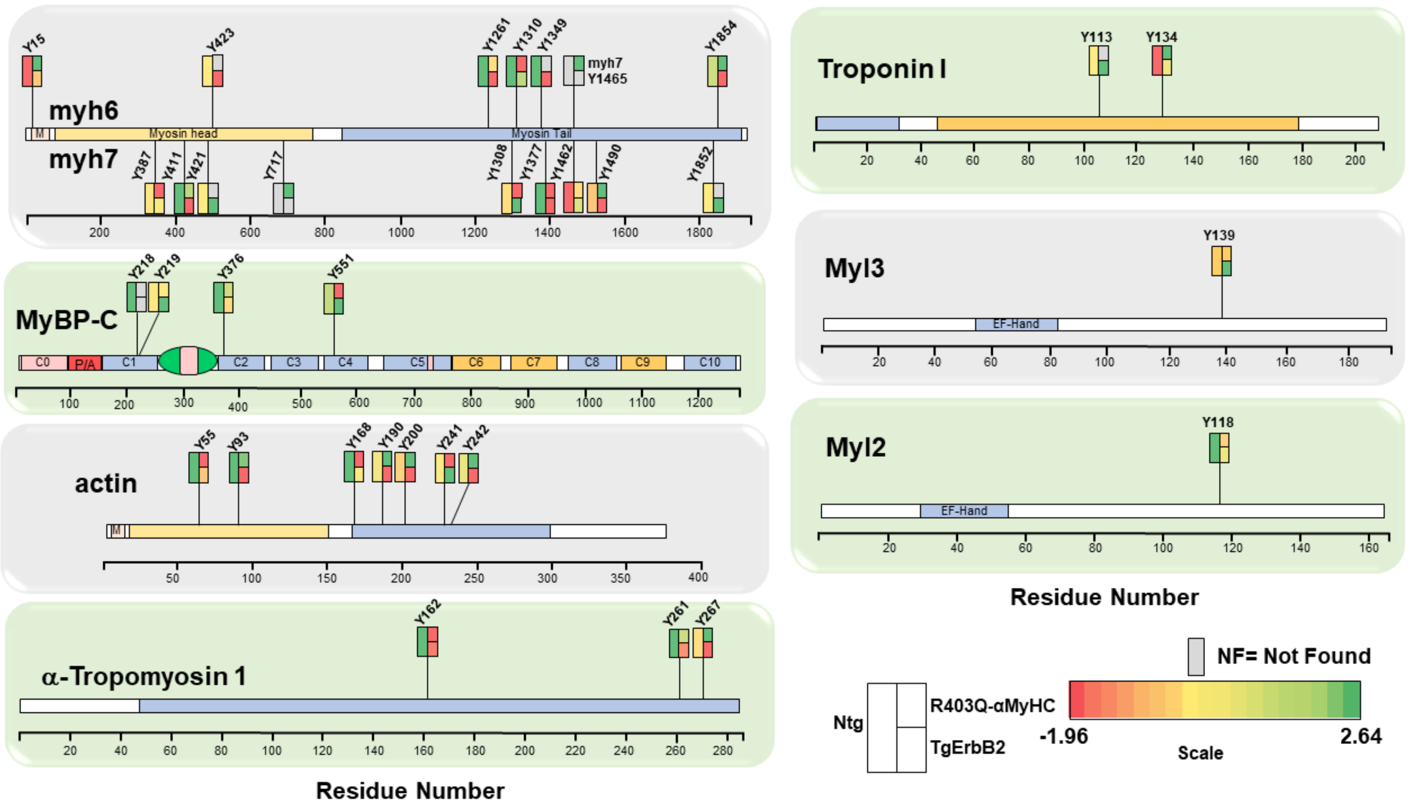

Supplementary Figure 2. Graphic Summary of Label-Free pTyr Site Profiling on Major Myofilaments Proteins. The figure displays the 34 pTyr sites detected on the major myofilaments myh6, myh7, MyBP-C, actin, tropomyosin, troponin I, MLC1 and MLC2, and its localization in relation to their functional domains or protein regions. A color-coded scale of Log2 of Ion Intensity of pTyr Peptides is on the lower right panel of the figure, and each square or rectangle corresponds to a genotype (i.e. Long rectangle Ntg, followed by stacked squares, gray color means that a particular site was not found on that genotype group).

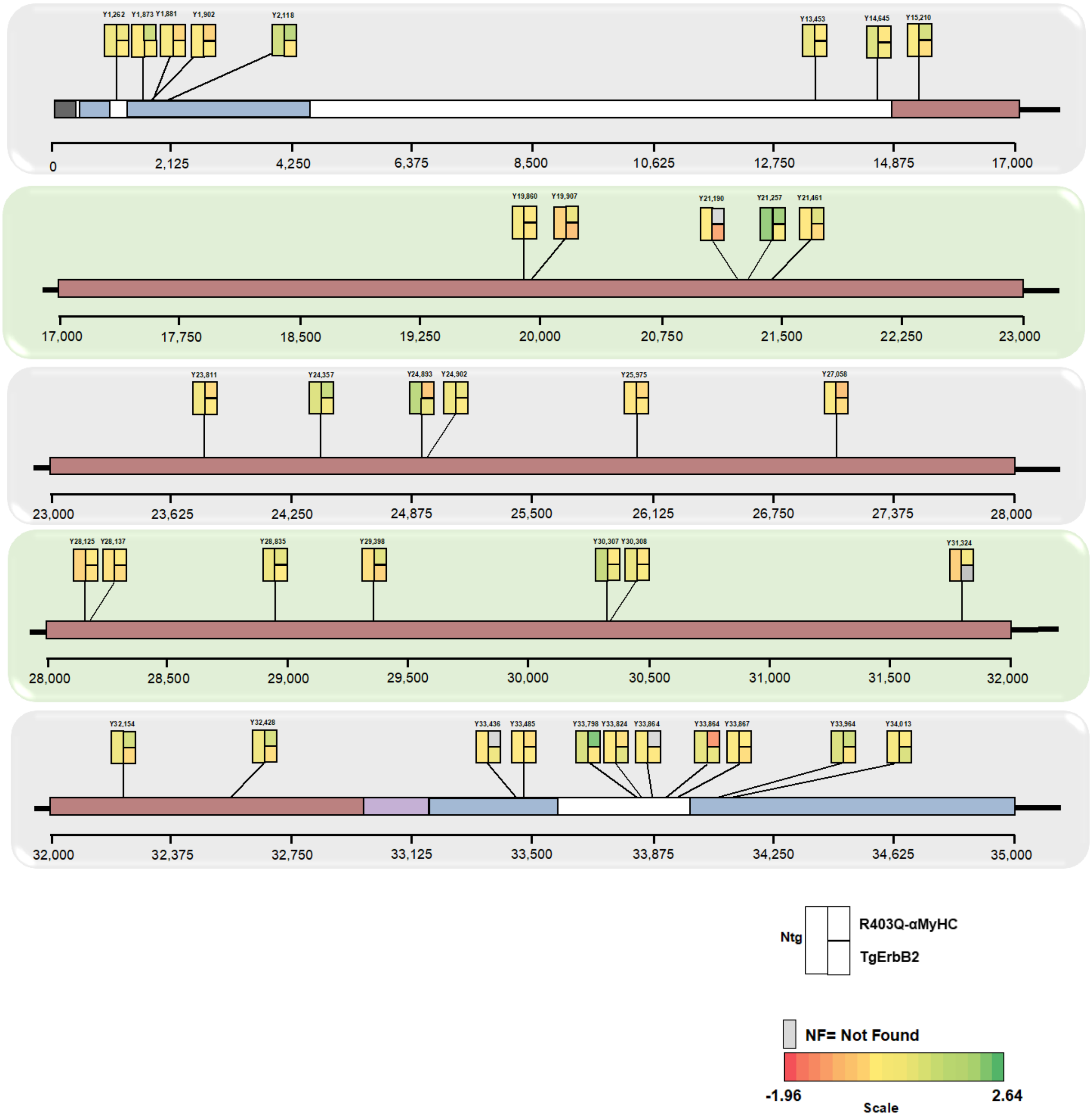

Supplementary Figure 3. Graphic Summary of Label-Free pTyr Site Profiling in titin. The figure displays the 36 pTyr sites detected in titin after pTyr immunoaffinity enrichment followed by MS/MS. A pTyr hotspot was found between the amino-acid 33,789 and 33,864. A color-coded scale of Log2 of Ion Intensity of pTyr Peptides is on the lower right panel of the figure, and each square or rectangle corresponds to a genotype (i.e. Long rectangle Ntg, followed by stacked squares, gray color means that a particular site was not found on that genotype group). To get an estimate of the relative abundance of pTyr sites, we applied MS1 extracted ion chromatograms using MaxQuant, especially on myofilaments in preparation for TMT labeling experiments on myofilaments enriched fraction of Ntg and TgErbB2 mice.

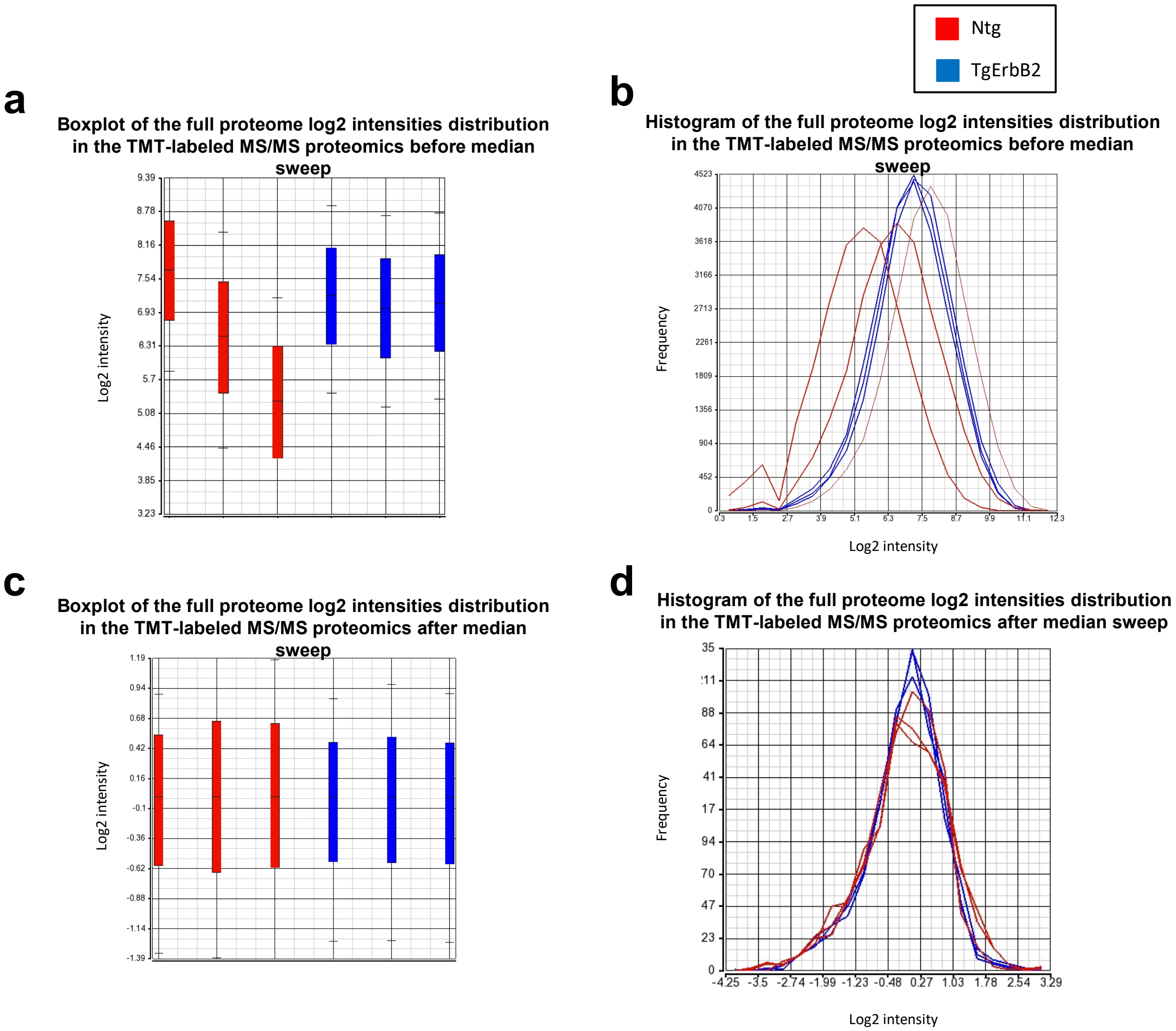

Supplementary Figure 4. Distribution of the protein expression intensities if the TMT-labeled MS/MS proteomics before and after median sweep normalization. a Boxplot showing the data distribution of the full proteome intensities from the TMT-labeled MS/MS proteomics before the median sweep. The y-axis represents the log2 intensity and each box represent one observation. **b** Histogram showing the data distribution of the full proteome log2 intensity of each observation obtained from the TMT-labeled MS/MS proteomics before the median sweep. Each line represent one observation and the values represented are the log2 intensity after median subtraction (y-axis) vs frequency. **c** Boxplot showing the data distribution of the full proteome intensities from the TMT-labeled MS/MS proteomics after the median sweep. The y-axis represents the log2 intensity and each box represent one observation. **d** Histogram showing the data distribution of the full proteome log2 intensity of each observation obtained from the TMT-labeled MS/MS proteomics after the median sweep. Each line represent one observation and the values represented are the log2 intensity after median subtraction (y-axis) vs frequency. The colors indicate the group to which each observation belong, Ntg (red), TgErbB2 (blue).

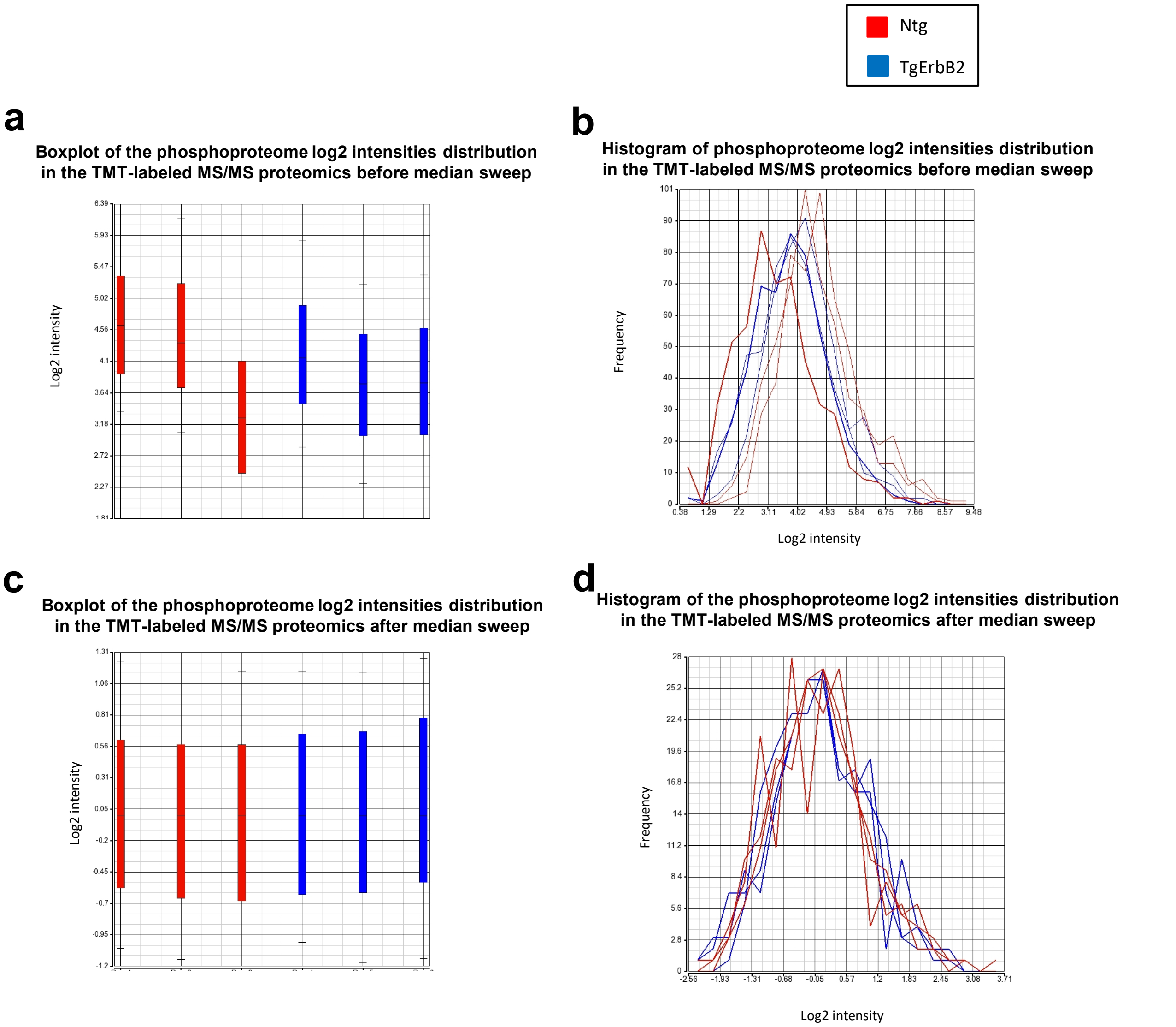

Supplementary Figure5. Distribution of the TMT phosphoproteome intensities data set before and after median sweep normalization. a Boxplot showing the data distribution of the phosphoproteome intensities from the TMT-labeled MS/MS proteomics before the median sweep. The y-axis represents the log2 intensity and each box represent one observation. **b** Histogram showing the data distribution of the phosphoproteome log2 intensity of each observation obtained from the TMT-labeled MS/MS proteomics before the median sweep. Each line represent one observation and the values represented are the log2 intensity after median subtraction (y-axis) vs frequency. **c** Boxplot showing the data distribution of the phosphoproteome intensities from the TMT-labeled MS/MS proteomics after the median sweep. The y-axis represents the log2 intensity and each box represent one observation. **d** Histogram showing the data distribution of the phosphoproteome log2 intensity of each observation obtained from the TMT-labeled MS/MS proteomics after the median sweep. Each line represent one observation and the values represented are the log2 intensity after median subtraction (y-axis) vs frequency. The colors indicate the group to which each observation belong, Ntg (red), TgErbB2 (blue).

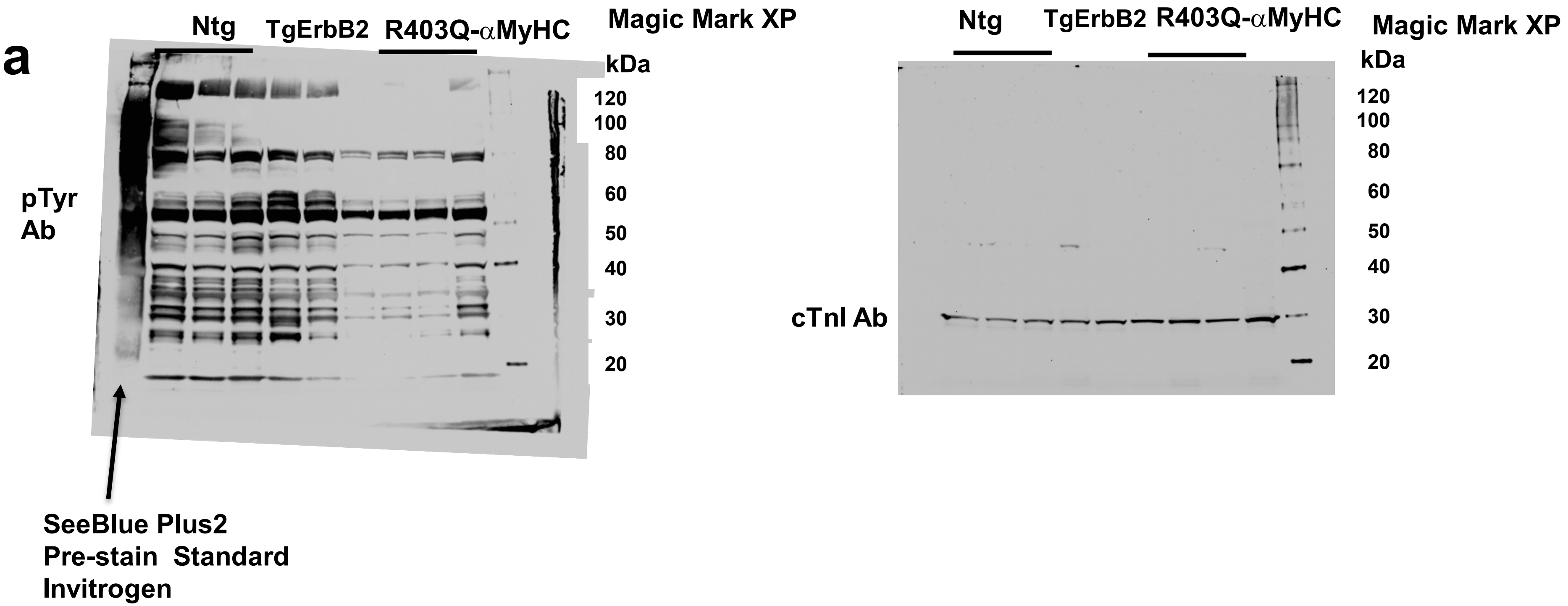

Supplemental Figure 6. Unprocessed and uncropped scans from Figure 1 a. Anti-Tyrosine antibody, the first lane has 6 ul of SeeBlue Plus 2 Pre-stained protein standard, the last lane contains 6 ul of Magic Mark XP. Duplicate samples were checked for control loading in a parallel gel using Anti-cTnI. SeeBlue Plus 2 might not be visible depending on the secondary antibody used. phospho-Tyrosine mouse mAb (pTyr-100, cat. No. 9411SCST), TnI Rabbit Ab (cat. No. 4002SCTS).

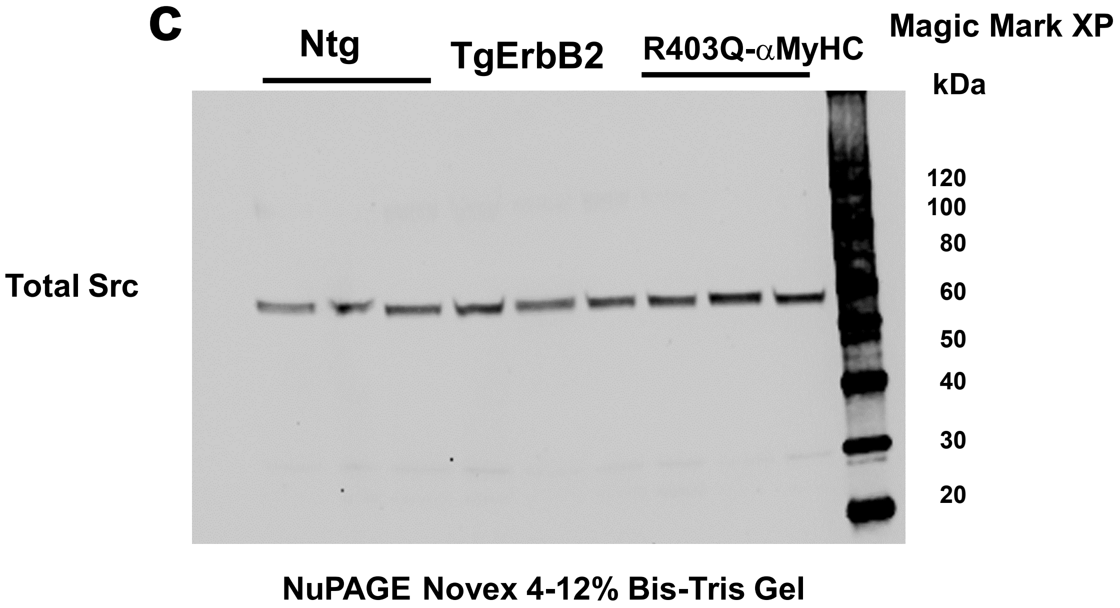

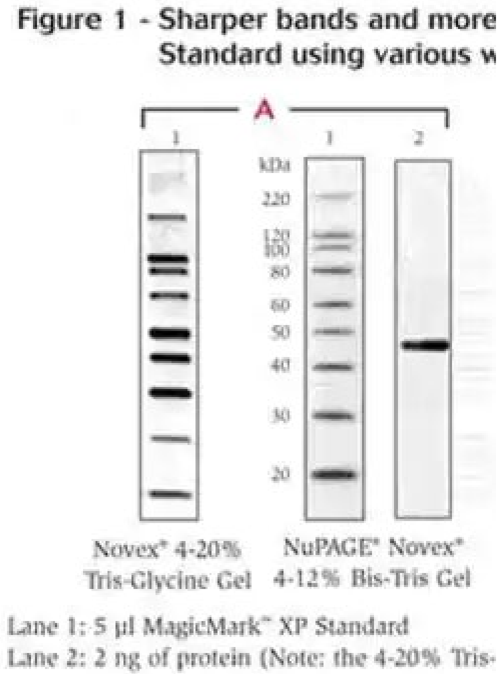

Supplemental Figure 6. Unprocessed and uncropped scans from Figure 1 c. Anti-src antibody, the last lane contains 6 ul of Magic Mark XP. Picture of Invitrogen website showing the pattern of Magic Mark XP in NuPAGE Novex 4-12% Bis-Tris Gel.), Src Rabbit Ab (cat. No.2108SCST).

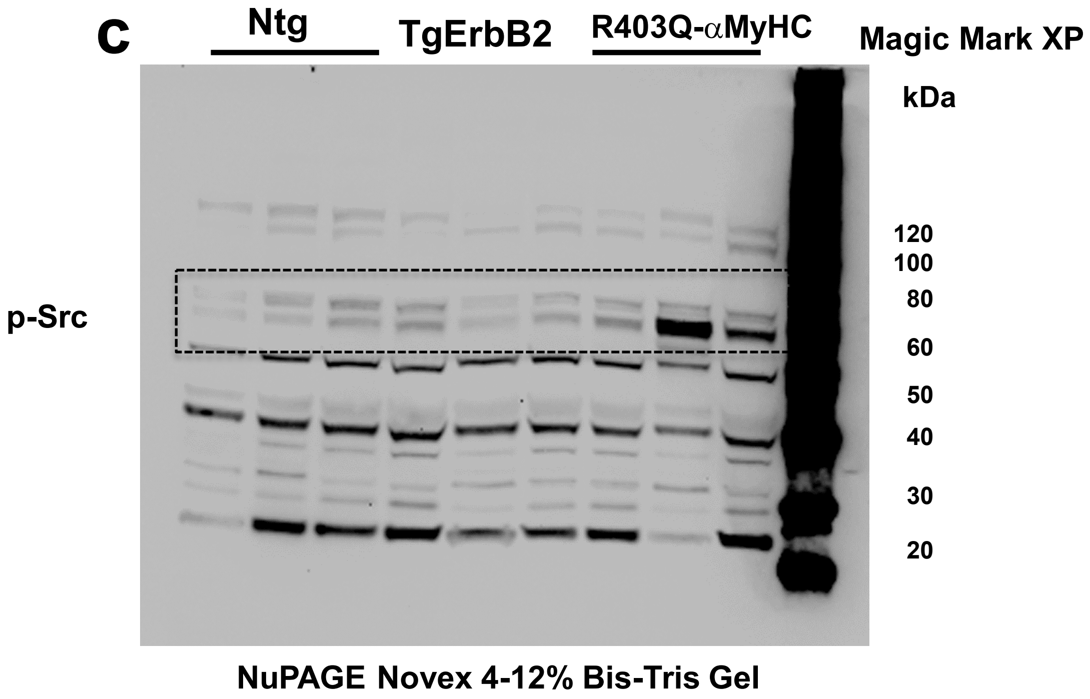

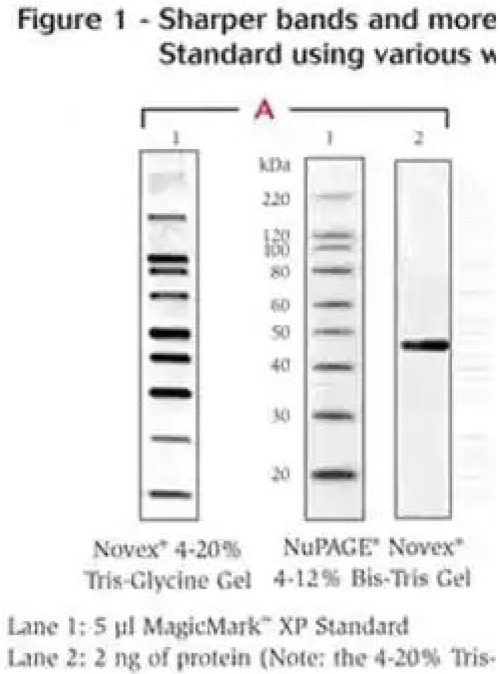

Supplemental Figure 6. Unprocessed and uncropped scans from Figure 1 c. Duplicate samples were checked for Anti-phospho src. The last lane contains 6 ul of Magic Mark XP. Picture of Invitrogen website showing the pattern of Magic Mark XP in NuPAGE Novex 4-12% Bis-Tris Gel. Unspecific bands were present; however, the specific signal was corroborated by the molecular weight of 60 kDa. A dashed square shows the cropped image used in Fig 1 of the main manuscript. Phopsho-Src Family (Tyr416) Rabbit mAb (D49G4, cat. No.6943T CST).

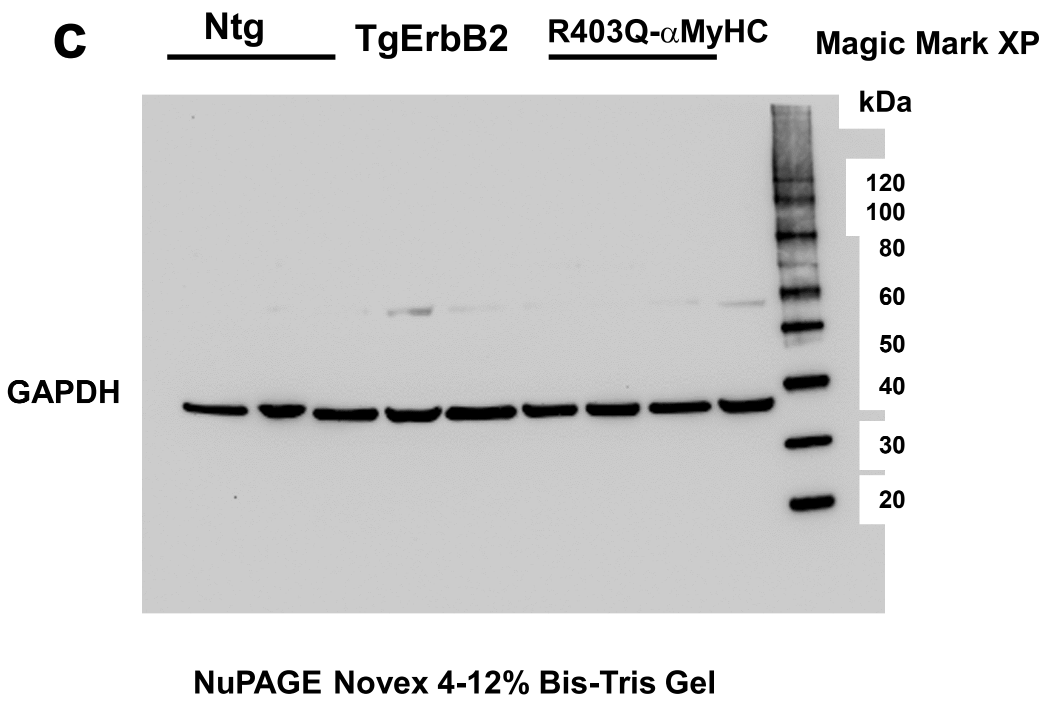

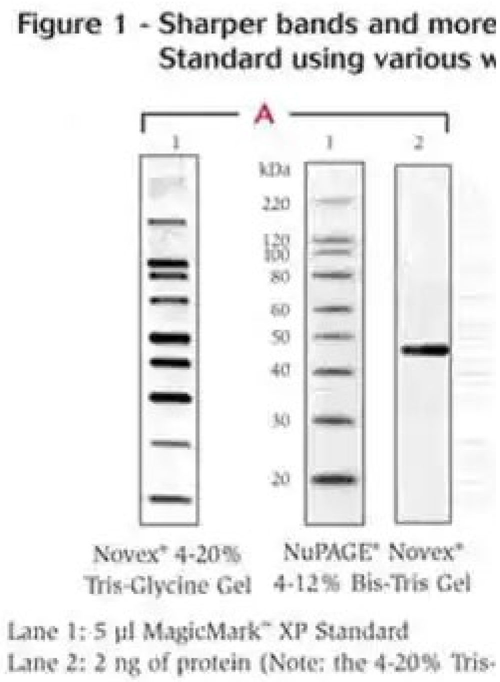

Supplemental Figure 6. Unprocessed and uncropped scans from Figure 1 c. Duplicate samples were checked for Anti-GAPDH for loading control. The last lane contains 6 ul of Magic Mark XP. Picture of Invitrogen website showing the pattern of Magic Mark XP in NuPAGE Novex 4-12% Bis-Tris Gel. GAPDH Rabbit Ab (14C10, cat. No. 2118S CST).
