# Supplementary material for: Alteration in tyrosine phosphorylation of cardiac proteome and EGFR pathway contribute to hypertrophic cardiomyopathy
Source: Commun Biol. 2022 Nov 15;5:1251. doi: 10.1038/s42003-022-04021-4 (PMC9666710; doi:10.1038/s42003-022-04021-4)
Supplement: Supplementary file 3 — Description of Additional Supplementary Files [file 42003_2022_4021_MOESM3_ESM.pdf]

Description of Additional Supplementary Files

File name: Supplementary Data 1
Description: Numerical source of graphs from Figure 1 and Figure 7.

File name: Supplementary Data 2
Description: Global whole heart TMT Flow-through Proteomics, Label-Free Phosphoproteomics, Imputed Phosphoproteomics (using KNN), and statistical analysis.

File name: Supplementary Data 3
Description: Cardiac Sarcomere TMT proteomics, phosphoproteomics, normalized phosphoproteomics, and statistical analysis.

File name: Supplementary Data 4
Description: Kinase Substrate Enrichment Analysis, Pathway Enrichment Analysis of identified modules.

File name: Supplementary Data 5
Description: Contractility and LV chamber dimensions by M- mode, and tissue doppler echocardiography.

File name: Supplementary Data 6
Description: Proteomics, comprehensive phosphoproteomics (Serine, Threonine, Tyrosine) in TgErbB2 and Tyrosine kinase receptor inhibition by AG-82.

File name: Supplementary Data 7
Description: Kinase Substrate Enrichment Analysis, Pathway Enrichment Analysis of AG-825 and treatment vehicle (sham).
