# Supplementary material for: Alteration in tyrosine phosphorylation of cardiac proteome and EGFR pathway contribute to hypertrophic cardiomyopathy
Source: Commun Biol. 2022 Nov 15;5:1251. doi: 10.1038/s42003-022-04021-4 (PMC9666710; doi:10.1038/s42003-022-04021-4)
Supplement: Supplementary file 8 — Supplementary Data 5 [file 42003_2022_4021_MOESM8_ESM.docx]

|  | Ntg Vehicle (Before treatment) | Ntg Vehicle (After treatment) | TgErbB2 Vehicle (Before treatment) | TgErbB2 Vehicle (After treatment) | Ntg AG-825 (Before treatment) | Ntg AG-825 (After treatment) | TgErbB2 AG-825 (Before treatment) | TgErbB2 AG-825 (After treatment) |
| --- | --- | --- | --- | --- | --- | --- | --- | --- |
| *n* | 4 | 4 | 4 | 4 | 4 | 4 | 5 | 5 |
| Left Ventricular Internal Diameter in Diastole, mm | 2.82±0.13 | 2.52±0.13 | 2.62±0.11 | 2.38±0.20 | 2.68±0.09 | 2.53±0.09 | 2.68±0.20 | 2.88±0.11 |
| Left Ventricular Internal Diameter in Systole, mm | 1.18±0.06 | 1.08±0.05 | 1.25±0.05 | 1.35±0.17 | 1.05±0.05 | 1.04±0.04 | 1.35±0.12 | 1.43±0.12 |
| Interventricular Septum in Diastole, mm | 1.02±0.04 | 1.08±0.07 | 1.90±0.04 | 2.08±0.05 | 1.00±0.03 | 1.06±0.03 | 1.79±0.11 | 1.80±0.14 |
| Left Ventricular posterior wall diameter in diastole, mm | 1.01±0.05 | 1.06±0.07 | 2.16±0.09 | 2.15±0.06 | 0.93±0.03 | 0.98±0.03 | 2.09±0.15 | 1.94±0.21 |
| Fractional Shortening (%) | 58.10±2.71 | 57.16±1.30 | 52.13±1.79 | 43.72±2.89 | 60.68±1.05 | 58.95±1.36 | 49.67±2.50 | 50.30±3.32* |
| Ejection Fraction (%) | 82.22±2.36 | 81.60±1.13 | 76.99±1.70 | 68.07±3.25 | 84.51±0.83 | 83.09±1.11 | 74.42±2.54 | 74.86±3.22* |
| Heart rate, beats/min | 716.25±17.00 | 710.00±12.42 | 628.75±34.84 | 633.75±38.43 | 715.00±13.69 | 708.75±22.30 | 635.00±14.83 | 625.00±13.78 |
| Relative Wall Thickness, mm | 0.72±0.06 | 0.85±0.09 | 1.67±0.13 | 1.83±0.10 | 0.70±0.04 | 0.78±0.04 | 1.60±0.17 | 1.35±0.15 |
| Left Ventricular Mass, mg | 97.13±5.64 | 90.29±8.47 | 295.00±8.14 | 295.70±41.57 | 83.13±3.49 | 83.27±5.05 | 283.94±36.29 | 293.12±46.42 |
| Early Diastolic Mitral Annular Velocity (E’), cm/s | 40.85±6.48 | 34.56±3.57 | 8.68±1.57 | 15.29±5.27 | 45.88±1.68 | 32.57±2.05 | 15.44±3.31 | 13.63±1.98 |
| Active Atrial Contraction in Late Diastole (A’), cm/s | 36.31±1.58 | 25.88±3.07 | 13.96±1.60 | 14.10±2.93 | 35.13±2.97 | 27.77±1.92 | 21.19±3.00 | 18.81±1.93 |
| E’/A’ ratio | 1.12±0.15 | 1.36±0.09 | 0.66±0.14 | 1.08±0.22 | 1.33±0.09 | 1.19±0.10 | 0.71±0.11 | 0.72±0.06 |
| Isovolumetric Relaxation Time, ms | 21.77±1.53 | 22.92±2.78 | 37.45±6.23 | 40.59±6.80 | 22.35±0.65 | 25.38±3.24 | 42.75±3.52 | 50.24±2.22 |

**Supplementary Table 4.** Contractility and LV chamber dimensions by motion mode and tissue Doppler echocardiography. Values represent averages and standard error of the mean of each measurement; n, no. of mice. *p-value " 0.05.
