# Supplementary material for: Alteration in tyrosine phosphorylation of cardiac proteome and EGFR pathway contribute to hypertrophic cardiomyopathy
Source: Commun Biol. 2022 Nov 15;5:1251. doi: 10.1038/s42003-022-04021-4 (PMC9666710; doi:10.1038/s42003-022-04021-4)
Supplement: Supplementary file 11 — Reporting Summary [file 42003_2022_4021_MOESM11_ESM.pdf]

nature portfolio

Corresponding author(s): Ramirez-Correa GA

Last updated by author(s): Sep 14, 2022

Reporting Summary

Nature Portfolio wishes to improve the reproducibility of the work that we publish. This form provides structure for consistency and transparency in reporting. For further information on Nature Portfolio policies, see our Editorial Policies and the Editorial Policy Checklist.

Statistics

For all statistical analyses, confirm that the following items are present in the figure legend, table legend, main text, or Methods section.

n/a	Confirmed	
☐	☒	The exact sample size (*n*) for each experimental group/condition, given as a discrete number and unit of measurement
☐	☒	A statement on whether measurements were taken from distinct samples or whether the same sample was measured repeatedly
☒	☐	The statistical test(s) used AND whether they are one- or two-sided<br>*Only common tests should be described solely by name; describe more complex techniques in the Methods section.*
☒	☐	A description of all covariates tested
☐	☒	A description of any assumptions or corrections, such as tests of normality and adjustment for multiple comparisons
☐	☒	A full description of the statistical parameters including central tendency (e.g. means) or other basic estimates (e.g. regression coefficient) AND variation (e.g. standard deviation) or associated estimates of uncertainty (e.g. confidence intervals)
☐	☒	For null hypothesis testing, the test statistic (e.g. *F*, *t*, *r*) with confidence intervals, effect sizes, degrees of freedom and *P* value noted<br>*Give P values as exact values whenever suitable.*
☒	☐	For Bayesian analysis, information on the choice of priors and Markov chain Monte Carlo settings
☐	☒	For hierarchical and complex designs, identification of the appropriate level for tests and full reporting of outcomes
☒	☐	Estimates of effect sizes (e.g. Cohen's *d*, Pearson's *r*), indicating how they were calculated

Our web collection on statistics for biologists contains articles on many of the points above.

Software and code

Policy information about availability of computer code

Data collection: Proteomic data collection was made with the manufacturer software for the equipment.

Data analysis: MaxQuant v1.5, was used for peptide matching against a mouse RefSeq database v78. R software v3.4.2, and Partek v7 was used for statistical analysis. For echorcardiography statistical analysis R software version 3.4.2 was used. For pathway analysis, Ingenuity Pathway Analysis Software and MATLAB. For myofibrilar disarray analysis we used CytoSpectre (web interface)

For manuscripts utilizing custom algorithms or software that are central to the research but not yet described in published literature, software must be made available to editors and reviewers. We strongly encourage code deposition in a community repository (e.g. GitHub). See the Nature Portfolio guidelines for submitting code & software for further information.

Data

Policy information about availability of data

All manuscripts must include a data availability statement. This statement should provide the following information, where applicable:

- Accession codes, unique identifiers, or web links for publicly available datasets
- A description of any restrictions on data availability
- For clinical datasets or third party data, please ensure that the statement adheres to our policy

The authors declare that the data supporting the findings is included in the main manuscript and in the supplementary data section.

Field-specific reporting

Please select the one below that is the best fit for your research. If you are not sure, read the appropriate sections before making your selection.

☒ Life sciences ☐ Behavioural & social sciences ☐ Ecological, evolutionary & environmental sciences

For a reference copy of the document with all sections, see nature.com/documents/nr-reporting-summary-flat.pdf

Life sciences study design

All studies must disclose on these points even when the disclosure is negative.

Sample size	For label-free proteomics 15 biological replicates were pooled into 3 technical replicates (five each). For revision we used flow-through and performed a 9-plex TMT to address full proteome. For TMT proteomics were 3 biological replicates and 1 technical replicate. For echocardiography, an 4 mice on each group were used, power test was used to calculate the sample size required for this experiment. For the comprehensive proteomics (pS/pT/pY) we did 13 more animals: Ntg-Vehilce (n=3), Ntg-AG825 (n=3), TgErbB2-Vehicle (n=3), TgErbB2-AG825 (n=4)
Data exclusions	For the mass spectrometry data analysis, one technical replicate was excluded due to the low signal adquired by the mass spectrometer. Also, peptides with low detection rate (absent in >50% of the samples) were excluded from the data analysis. No other data was excluded.
Replication	Principal component analysis, and unsupervised hierarchical clustering were used to measure the reproducibility of the results obtained by mass spectrometry. The differences in pTyr profiling were evaluated by mas spectrometry and western blot. Echocardiography and microscopy findings correlate with previous reports found in the literature. New comprehensive phospho-proteomics (pS/pT/pY) confirmed the previous findings related to involved diseases pathwats
Randomization	Each experimental group was defined by the mouse genotype. The groups were defined as cardiac ErbB2 overexpression mice, R403Q-aMyHC and non-transgenic. The treatment of Vehilce or AG-825 for NTg or TgErbB2 was randomized and then protoemics performed on samples
Blinding	Echocardiography and and microscopy imaging studies, were performed double blinded. Proteomic studies were non-blinded.

Reporting for specific materials, systems and methods

We require information from authors about some types of materials, experimental systems and methods used in many studies. Here, indicate whether each material, system or method listed is relevant to your study. If you are not sure if a list item applies to your research, read the appropriate section before selecting a response.

Materials & experimental systems

n/a	Involved in the study	
☐	☒	Antibodies
☒	☐	Eukaryotic cell lines
☒	☐	Palaeontology and archaeology
☐	☒	Animals and other organisms
☒	☐	Human research participants
☒	☐	Clinical data
☒	☐	Dual use research of concern

Methods

n/a	Involved in the study	
☒	☐	ChIP-seq
☒	☐	Flow cytometry
☒	☐	MRI-based neuroimaging

Antibodies

Antibodies used	Phospho-Tyrosine Mouse mAb (pTyr-100, Cell signaling, #9411), Src Antibody (Cell signaling, #2108), Phospho-Src Family (Tyr416) (D49G4) Rabbit mAb (Cell signaling, #6943), Anti-cTnI (Spectral Diagnostics, MA-1040).
Validation	Phospho-Tyrosine Mouse mAb (P-Tyr-100) #9411: Tested on extracts from Jurkat cells treated with 1 mM pervanadate for 30 minutes prior to lysis (Western blot available on manufacturer website).<br>Src Antibody #2108: Tested on extracts from various cell lines (Western blot available on manufacturer website).<br>Phospho-Src Family (Tyr416) (D49G4) Rabbit mAb #6943: Tested on extracts NIH/3T3 cells, serum-starved or treated with human Platelet-Derived Growth Factor (Western blot available on manufacturer website).<br>Anti-cTnI (Spectral Diagnostics, MA-1040): Circ. Res. 1999;84:9-20.

Animals and other organisms

Policy information about studies involving animals; ARRIVE guidelines recommended for reporting animal research

Laboratory animals	The animals used were Mus musculus, both males and females, the TgErbB2 were originally constructed from the strain B6SJLF1/J.

Laboratory animals	The R403Q-aMyHC mice were originally constructed from the strain C57/B16. The animal samples for proteomic analysis were obtained at 6-9 months old. Non-transgenic litter mates were used as control.
Wild animals	This study did not include wild animals
Field-collected samples	This study did not analyzed field-collected samples.
Ethics oversight	All protocols were performed following the "Guide for the Use and Care of Laboratory Animals" Published by the National Institute of Health and the Institutional Animal Care and Use Comitee's approval.

Note that full information on the approval of the study protocol must also be provided in the manuscript.
